# Physio-Morphological Assessment and Multi-Omics Analysis of *Flammulina filiformis* Mycelial Ageing

**DOI:** 10.3390/jof12090708

**Published:** 2026-09-21

**Authors:** Keming Zhu, Zenan Huang, Waner Lv, Yingjie Liu, Zhiying Lin, Zhiyuan Wang, Haoran Xu, Chengzhen Gu, Shujing Sun

**Affiliations:** 1 College of Life Sciences, Fujian Agriculture and Forestry University, Fuzhou 350002, China; 52405044012@fafu.edu.cn (K.Z.); zhard22@163.com (Z.H.); 12505014045@fafu.edu.cn (W.L.); 52505044020@fafu.edu.cn (Y.L.); 52505044028@fafu.edu.cn (Z.L.); 52505044006@fafu.edu.cn (Z.W.); 3235411055@stu.fafu.edu.cn (H.X.); 2 Gutian Edible Fungi Research Institute, Fujian Agriculture and Forestry University, Ningde 352200, China

**Keywords:** *Flammulina filiformis*, mycelial ageing, metabolomics, mycelium-associated bacteria, correlation analysis

## Abstract

*Flammulina filiformis* is one of the most important commercially cultivated species, and the assessment of strain quality is of paramount importance. The analysis measured physiological-biochemical indicators, morphological indicators, combined non-targeted metabolomics, and microbiomics in *F. filiformis* strains of different ages. The results indicate that, as the culture age increased, ergosterol content and catalase (CAT) activity declined continuously. The morphology of the mycelia gradually deteriorated, and yield continued to decrease. The relative content of lipids and lipid-like molecules decreased overall, while the proportion of organic acids and their derivatives increased; the oxidative phosphorylation pathway continued to be significantly enriched. The richness and diversity of the mycelium-associated bacterial community in the mycelia decreased significantly, and the microbial community structure changed. The community was dominated by the genus *Acinetobacter* from 15 to 60 days, whereas the genus *Serratia* was predominant in 90 days group. Correlation analysis revealed that the core differential signature metabolites MG 14:1_0:0_0:0, aspartic acid-threonine-aspartamide, and nicotinamide adenine dinucleotide (NAD^+^) were significantly correlated with multiple physiological-biochemical indicators. N-arachidonyl glycine showed significant correlations with 21 core bacterial genera; the dominant genera *Acinetobacter* and *Serratia* exhibited strong correlations with membrane homeostasis, core indicators of oxidative damage, and signature metabolites. These results indicate that the mycelial vitality of *F. filiformis* remains good within the first 15 days of cultivation, with significant ageing occurring after 60 days. These findings provide a theoretical basis for optimizing fungal strain preservation techniques and regulating mycelial ageing.

## 1. Introduction

*Flammulina filiformis* is one of the most important varieties of edible fungi cultivated in industrial-scale facilities worldwide and is rich in nutrients and highly popular with consumers [1]. The quality of spawn is a key factor in ensuring stable production. However, large-scale production currently relies heavily on long-term subculturing and preservation of spawn [2], which makes it highly susceptible to mycelial ageing and decline. This directly leads to a reduction in spawn viability [3,4], unstable cultivation yields [3,5] and a deterioration in product quality [6,7].

The ageing of fungal mycelia is commonly associated with an imbalance in the antioxidant system [8,9,10], damage to cell membrane structure [9,11], and disruption of intracellular nutrient metabolism [12], alongside common phenotypic traits such as morphological abnormalities in colonies and a decline in fruiting body production [5,6,7,13]. As the harvest time of fruiting bodies is delayed, the balance between reactive oxygen species (ROS) and antioxidants in *Agaricus bisporus* is disrupted, causing extensive ageing of edible fungal tissues. During this process, the malondialdehyde (MDA) content increased [10]. Transcriptome analysis of four developmental stages of *Agaricus bisporus* fruiting bodies revealed that a set of significantly differentially expressed genes were enriched in various metabolic pathways [14], suggesting that the ageing process in edible fungi may be associated with the downregulation of specific metabolic pathways.

When different bacteria are co-cultured with *Schizophyllum commune*, they induce a series of significant morphological changes in the fungal mycelia, including growth inhibition, mycelial proliferation, pigment production, and impaired fruiting body formation. This suggests that bacteria can profoundly influence fungal growth and development, which may involve physiological changes associated with ageing [15]. An analysis of the bacterial community structure of *Pleurotus eryngii* at different growth stages was conducted, and multiple bacterial strains capable of promoting mycelial growth and increasing fruiting body yield were isolated, demonstrating that the dynamics of microbial communities have a critical impact on the growth and development of edible fungi [16]. In this study, the bacterial community of the above types are defined as mycelium-associated bacteria.

In this study, core differentially expressed metabolites, key metabolic pathways and succession patterns in the mycelium-associated bacterial community during the ageing process of *F. filiformis* mycelial ageing were systematically analyzed, using non-targeted metabolomics and microbiomics. The rule of synergy will be revealed through metabolism and microbial interactions during mycelial ageing. The results will provide a theoretical basis for optimizing *F. filiformis* strain preservation techniques and regulating mycelial ageing.

## 2. Materials and Methods

### 2.1. Obtaining Samples

Potato dextrose agar (PDA) medium was purchased from Qingdao Hi-tech Industrial Park Hope Bio-technology Co., Ltd. (Qingdao, China).

The cultivation formula consisted of 60% sawdust, 20% cottonseed hulls, 15% wheat bran, 2% lime, 1.5% gypsum powder, 1.5% superphosphate, and the moisture content of 60–65%. Cultivation process management refers to the methods of Liu et al. [17].

The *F. filiformis* strain was preserved by the Team for Innovation and Metabolic Engineering of Special Edible Fungi at Fujian Agriculture and Forestry University. The strains used in this experiment must be activated by culturing them for 7 days at 25 °C on PDA for two generations. The samples were cultured on PDA medium at 25 °C, and mycelial samples were collected at 15, 30, 45, 60, 75, and 90 days. The mycelia were scraped under sterile conditions and stored at –80 °C for future use. The mycelia and fruiting bodies of the following experiments were taken from 6 replicates of different ages.

### 2.2. Determination of Physiological-Biochemical Indicators

The levels of soluble protein, soluble sugars, ergosterol, MDA and free amino acids were determined in accordance with relevant literature [18,19,20,21,22], while CAT activity was measured using a commercial assay kit (purchased from Sangon Biotech (Shanghai) Co., Ltd., Shanghai, China). The microscopic structure of the mycelia was examined using the method described by Cao et al. [23].

### 2.3. Metabolomic Analysis

A solid sample of 50 mg was taken, and metabolites were extracted using 400 µL of extraction solution (methanol:water = 4:1 (*v*:*v*)) containing 0.02 mg/mL of an internal standard (L-2-chlorophenylalanine (0.02 mg/mL)). The sample solution was ground while frozen for 6 min (−10 °C, 50 Hz), and then low-temperature ultrasonic extraction was performed for 30 min (5 °C, 40 kHz). The sample was allowed to stand at −20 °C for 30 min, centrifuged for 15 min (4 °C, 13,000 g), and the supernatant was transferred to an injection vial for analysis. An equal volume of metabolites from all samples was mixed to prepare a quality control (QC) sample. During instrumental analysis, one QC sample was inserted for every eight samples to assess the reproducibility of the entire analytical process.

LC-MS/MS analysis of the samples was performed using ultra-high-performance liquid chromatography coupled with Fourier transform mass spectrometry (UHPLC-Q Exactive HF-X, Thermo Fisher Scientific (China) Co., Ltd., Shanghai, China). Take 3 μL of the sample, separate it using the ACQUITY UPLC HSS T3 column (100 mm × 2.1 mm i.d., 1.8 µm), and then analyze it by mass spectrometry. Mobile phase A consisted of 95% water + 5% acetonitrile (containing 0.1% formic acid), and mobile phase B consisted of 47.5% acetonitrile + 47.5% isopropanol + 5% water (containing 0.1% formic acid). The flow rate was 0.40 mL/min, and the column temperature was 40 °C.

Mass spectrometry analysis was performed using dual-mode scanning (positive and negative ions) with a mass scan range of 70–1050 *m*/*z*. The ion source parameters were set as follows: sheath gas flow rate 50 psi, auxiliary gas flow rate 13 psi, and auxiliary gas heater temperature 425 °C; ion spray voltage: +3500 V in positive ion mode and –3500 V in negative ion mode. Mass spectrometer resolution: 60,000 for the first stage and 7500 for the second stage. Data acquisition was performed in data-dependent acquisition (DDA) mode.

### 2.4. DNA Extraction and Sequencing

DNA was extracted from mycelia according to the instructions of the E.Z.N.A.^®^ Soil DNA Kit (Omega Bio-Tek, Norcross, GA, USA); 1% agarose gel electrophoresis was used to analyze DNA integrity. The PCR products were purified using the TruSeq™ DNA Sample Prep Kit (Illumina (China) Scientific Co., Ltd., Shanghai, China), followed by library preparation and sequencing on the Illumina NextSeq 2000 platform (Illumina (China) Scientific Co., Ltd., Shanghai, China).

### 2.5. Data Analysis

The raw LC-MS data were imported into the metabolomics analysis software Progenesis QI v3.0 (Waters Corporation, Milford, MA, USA) for baseline filtering, peak identification, integration, retention time correction, and peak alignment, ultimately yielding a data matrix containing retention times, mass-to-charge ratios, and peak intensities. MS and MS/MS mass spectrometry data were matched against the public metabolomics databases human metabolome database (HMDB) (http://www.hmdb.ca, accessed on 5 April 2025) and Metlin (http://metlin.scripps.edu, accessed on 15 April 2025), as well as a self-constructed database on the Majorbio Cloud platform (https://web.archive.org/web/20250214233848/https://www.majorbio.com/, accessed on 20 April 2025), to obtain metabolite information (All lipids were named according to the standardized nomenclature guidelines published by Liebisch et al. [24], and were putatively annotated (MSI Level 2) via MS/MS matching to HMDB, Metlin, and an Majorbio in-house library; chain compositions, sn-positions, and double-bond assignments were not verified against standards, and given acyl migration risk in lysophospholipids, results should be read at the species level with caution) Via MS/MS matching to HMDB, Metlin, and a Majorbio in-house library; chain compositions, sn-positions, and double-bond assignments were not verified against standards, and given acyl migration risk in lysophospholipids, results should be read at the species level with caution. Missing values in the data matrix were removed using the 80% rule; the data were normalized by sum, and variables with a relative standard deviation (RSD) > 30% in QC samples were excluded. Principal component analysis (PCA) was performed using the ropls package v.1.38.0 in R program v.4.5.1. The criteria for identifying differentially expressed metabolites were variable importance in projection (VIP) > 1 and *p* < 0.05.

For microbiomics analysis, the fastp software (http://github.com/OpenGene/fastp, version 0.19.6, accessed on 18 October 2025) [25] was used to perform quality control on the raw paired-end sequencing reads, and FLASH software (http://cbcb.umd.edu/software/flash, version 1.2.11, accessed on 20 October 2025) [26] was used for assembly. The DADA2 plugin [27] within the Qiime2 workflow [28] and the DADA2 plugin [27] to perform noise reduction on the optimized sequences obtained after quality control and assembly; the sequences resulting from this process are referred to as amplified sequence variants (ASVs). Sequences annotated to chloroplasts and mitochondria were removed from all samples, and species-level taxonomic analysis of the ASVs was performed using the Naive Bayes classifier in Qiime2, based on the Sliva 16S rRNA gene database (v 138). The Mothur (v 1.30) software [29] was used to calculate alpha diversity indices (Chao 1, Shannon, Simpson, and Pd indices); principal component analysis (PCA) was employed to examine differences between samples, and the Adonis non-parametric test was used to determine whether these differences were significant; Python 2.7 was used to generate between-group community composition plots and between-group Upset plots. Beta diversity PCA analysis.

All data analysis was conducted on the MajorBio Cloud Platform (https://web.archive.org/web/20250214233848/https://www.majorbio.com/, accessed on 20 April 2025) [30]. Significance analysis was performed using IBM SPSS Statistics 23 (*p* < 0.05).

## 3. Results

### 3.1. Analysis of Physiological-Biochemical Indicators

The content of soluble proteins and soluble sugars reflects changes in intracellular storage compounds and the baseline nutritional metabolic status of mycelia [9,31]. The levels of soluble protein and soluble sugars showed an initial increase followed by a decrease as culture age increased. Among these, soluble protein content was higher at 45, 60, and 75 days, while soluble sugars content was higher at 45 and 60 days and significantly higher than in the other groups (Table S1).

Ergosterol is an important component of fungal cell membranes; changes in its concentration reflect changes in the structural stability and integrity of the cell membrane [32]. CAT is an important antioxidant defense enzyme; changes in its activity reflect the mycelial ability to scavenge reactive oxygen species and maintain redox homeostasis [4]. MDA is a major product of lipid peroxidation, and its accumulation can be used to assess the level of oxidative damage to cell membranes [33]. The levels of ergosterol and CAT activity showed a decreasing trend, with the highest values observed at 15 days (1.08 mg·g^−1^ and 2.79 U·g^−1^, respectively) and the lowest at 90 days (0.54 mg·g^−1^ and 0.62 U·g^−1^, respectively), representing decreases of 50% and 77.78%, respectively. MDA levels were lowest at 90 days and were significantly lower than those at 30, 45, and 60 days (*p* < 0.05) (Table S1).

Changes in the composition of free amino acids can be used to assess dynamic changes in nitrogen metabolism and protein metabolism [34,35]. Most amino acids showed an initial increase followed by a decrease as culture age increased; glutamine and cysteine showed an initial decrease followed by an increase; and aspartic acid and glutamic acid showed no clear trend. Tyrosine showed an upward trend. The contents of the four amino acids—leucine, valine, threonine, and proline were the highest at 30 days, with 1.150 mg·g^−1^, 0.888 mg·g^−1^, 0.711 mg·g^−1^, and 0.547 mg·g^−1^, respectively; with decrease rates of 82.43%, 79.84%, 80.59%, and 72.76%, respectively, at 90 days. The content of tyrosine at 15 days was the lowest with 0.727 mg·g^−1^ and reached 1.537 mg·g^−1^ at 90 days, with an increase of 111.4% (Table S2).

### 3.2. Analysis of Mycelial Morphological Characteristics and Fruiting Performance

**Figure 1:**
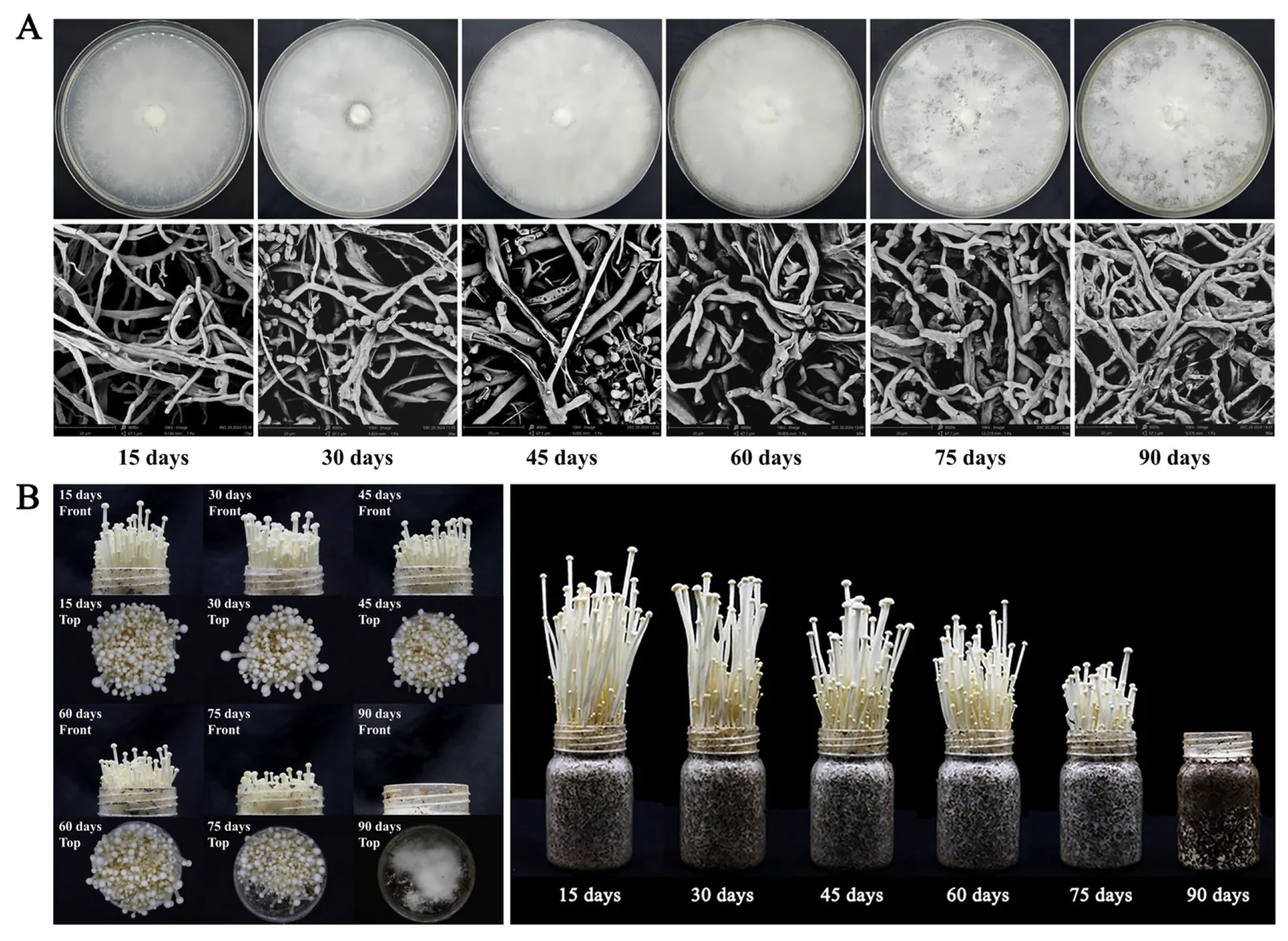
Characteristics of mycelia and fruiting bodies at different growth stages. (**A**) Macromorphology and ultrastructure of mycelia (4000×); (**B**) Growth status and maturity comparison of fruiting bodies.

Significant differences in mycelial morphology were observed across different growth stages. At 15 days, the mycelia were uniform and pure white, with numerous branches and neat colony margins. At 30 days, mycelial growth was vigorous, and abundant spores were produced. At 45 days, colony uniformity decreased; the mycelia became uneven and swollen, and spore production declined. At 60 days, colonies were shriveled, mycelia became distorted, and sporulation was nearly absent. At 75 days, colonies showed localized invaginations, and mycelia began to undergo degradation. At 90 days, the areas of invagination expanded, and mycelial degradation intensified (Figure 1A). With increasing culture age, the number of clamp connections decreased, while spore production capacity, aerial mycelial strength, and mycelial density exhibited an initial increase followed by a decline; however, the mycelial branching angle increased progressively (Table S3).

Stem length, fruit weight, and biological efficiency showed a downward trend as culture age increased (Table S4). There were a large number of edible fungi buds, which exhibited the best fruiting performance (Figure 1B), with the longest stem length of 136.10 mm, the highest fruiting body weight per bottle of 53.27 g, and the highest biological efficiency of 60.88% at 15 days (Table S4). The fruiting performance of 30, 45, and 60 days declined in that order. However, all produced fruiting bodies (Figure 1B). At 75 days, the stem length decreased to 70.43 mm, a 48.25% reduction compared to 15 days, while the fruiting bodies’ weight per bottle was 42.87 g, a decrease of 19.52% (Table S4). The fruiting bodies did not form at 90 days, and the bottle mouth was covered with a large number of aerial mycelia (Figure 1B). No clear pattern of change was observed in stem thickness, cap diameter or cap height (Table S4).

Based on the overall physiological alteration trend during ageing, four representative time-points, including early-stage 15 days, middle-stage 30 days, mid-late-stage 60 days, and late-ageing-stage 90 days, were selected for metabolomic and microbiomic profiling.

### 3.3. Metabolomic Analysis

#### 3.3.1. Analysis of Metabolites Across Groups

**Figure 2:**
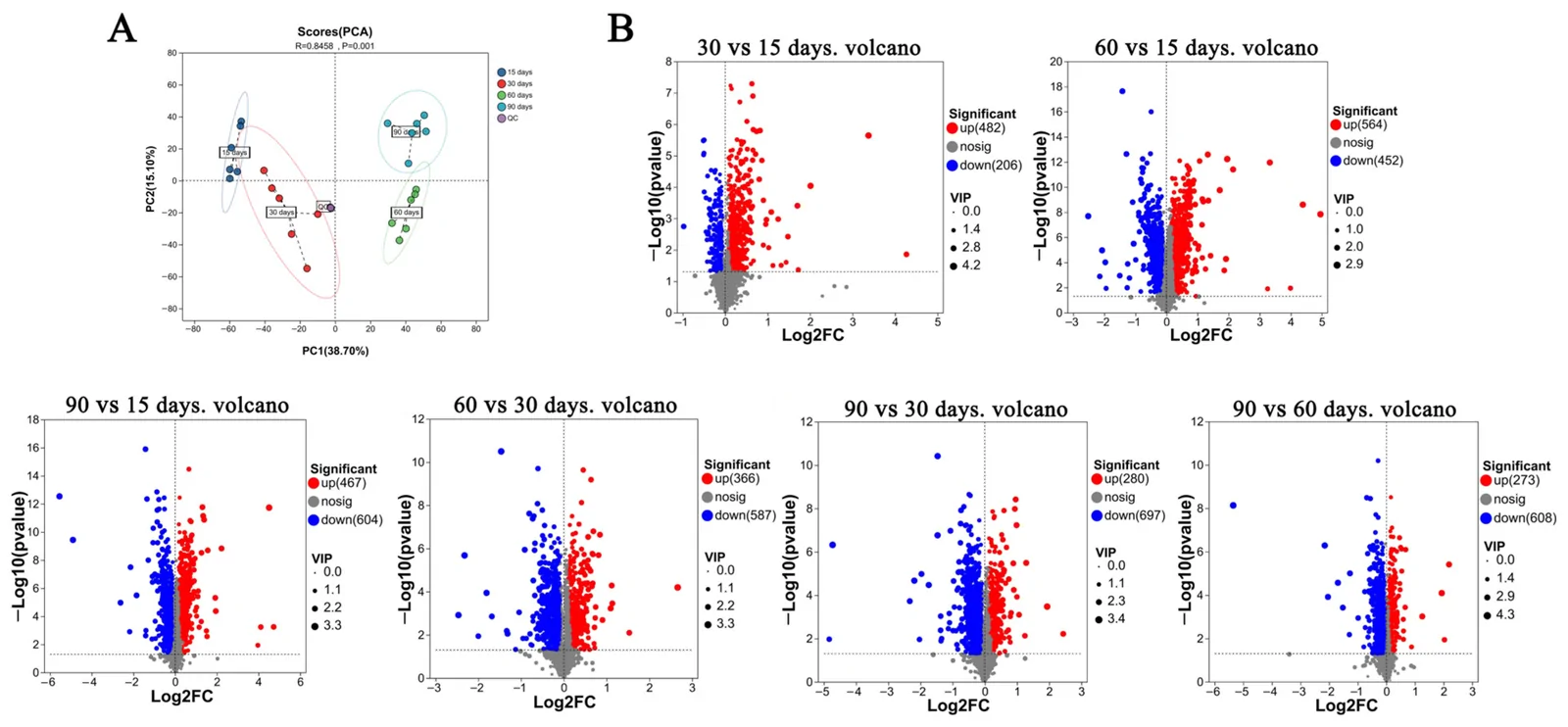
Overall distribution and differential analysis of metabolites. (**A**) Principal component analysis, (**B**) Volcanic map of inter-group differential metabolites.

To elucidate dynamic changes in the metabolic profiles of *F. filiformis* mycelia at different ages, non-targeted metabolomic analysis of mycelial samples from 15, 30, 60, and 90 days, which exhibited significant differences in growth phenotypes, was employed. A total of 2055 metabolites were identified; following annotation via the KEGG database, 974 metabolites were confirmed. PCA analysis revealed that the first and second principal components collectively accounted for 53.80% of the variance. The samples from the four growth-stage groups were clearly separated from one another (Adonis test, *p* = 0.001) (Figure 2A) and clustered within their respective groups. A volcano plot of differentially expressed metabolites was generated (Figure 2B), with screening criteria set at VIP > 1 and *p* < 0.05. The number of differentially expressed metabolites in each comparison group was as follows: 482 metabolites were up-regulated and 206 were down-regulated in the 30 vs. 15 days; 564 were up-regulated and 452 were down-regulated in the 60 vs. 15 days; 467 were up-regulated and 604 were down-regulated in the 90 vs. 15 days; 366 were up-regulated and 587 were down-regulated in the 60 vs. 30 days; 280 were up-regulated and 697 were down-regulated in the 90 vs. 30 days; 273 were up-regulated and 608 were down-regulated in the 90 vs. 60 days. The overall trend in differential metabolites shifted from upregulation to downregulation as the culture age increased.

**Figure 3:**
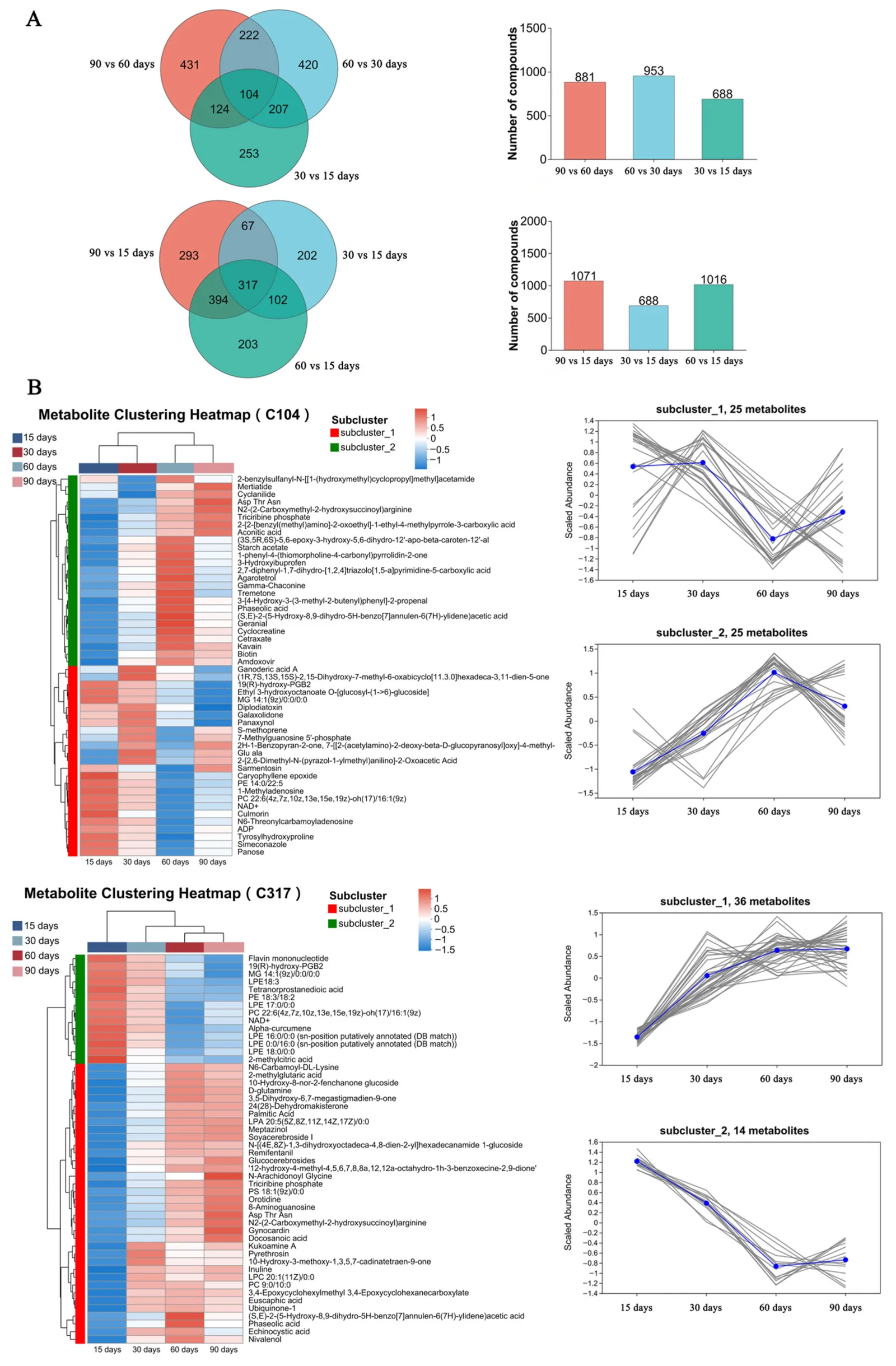
Screening and expression patterns of core differential metabolites. (**A**) Venn diagram of differential metabolite sets; (**B**) Clustering heat map and trends in changes across different clusters. In the trend plots, grey lines indicate normalized abundance trajectories of individual metabolites, and the blue line represents the average trend of metabolites in each subcluster.

Analysis of the Venn diagrams as shown in Figure 3A revealed that the comparisons between adjacent stages (30 vs. 15 days, 60 vs. 30 days, and 90 vs. 60 days) contained a total of 104 shared differential metabolites. These metabolites exhibited significant differences across various time intervals, with their significance levels reflecting the time intervals with the greatest variation. They can be used to assess trends in the overall metabolic profile of the mycelia and to identify inflection points in these trends; these metabolites were defined as the core metabolite set Core104 (C104). The three comparison groups using 15 days as a control (30 vs. 15 days, 60 vs. 15 days, and 90 vs. 15 days) revealed a total of 317 shared differential metabolites. These metabolites reflected the degree of difference between 30, 60, 90, and 15 days, indicating a trend in mycelial metabolic profiles with increasing age. These 317 metabolites were defined as the core metabolite set Core317 (C317). Cluster analysis of the core metabolites in both Core104 and Core317 indicated that 60 days represented an inflection point in metabolic profile changes.

#### 3.3.2. Dynamic Analysis of Marker Metabolites

To further investigate trends in metabolite changes showing significant differences, cluster analysis was performed on the core metabolite sets C104 and C317, as shown in Figure 3B.

As can be seen from Figure 3B, the clustering results for C104 were divided into two clusters, both of which exhibit an inflection point at 60 days, suggesting that 60 days may be the culture age at which changes in mycelial metabolic characteristics occur. As the culture age increases, metabolites showing an upward trend include aspartic acid-threonine-aspartamide and aconitic acid; those showing an initial rise followed by a decline include ganoderic acid A and biotin; and those showing an initial decline followed by a rise include caryophyllene epoxide, NAD^+^, 1-methyladenosine, PE 14:0_22:5, N6-threonylcarbamoyladenosine, ADP, and panose; metabolites showing a decreasing trend included MG 14:1_0:0_0:0 and ethyl 3-hydroxyoctanoate O-glucosyl-(1->6)-glucoside. The clustering results for C317 were divided into two clusters. The clustering pattern reflected trends in metabolites in the control sample (15 days); the two clusters exhibited upward and downward trends, respectively, but both tended to stabilize at 60 days. As the culture age increases, metabolites showing an upward trend include glucocerebroside, orotidine, aspartic acid-threonine-aspartamide, docosanoic acid, LPS 18:1, N-arachidonyl glycine metabolites exhibiting an initial rise followed by a decline included 2-methylglutaric acid, D-glutamine, palmitic acid, and ubiquinone-1; metabolites exhibiting an initial decline followed by a rise included NAD^+^, α-curcumene, LPE 16:0, and LPE 18:0; metabolites showing a decreasing trend include MG 14:1_0:0_0:0, flavin mononucleotide, PE 18:3_18:2, LPE 18:3, and 2-methylcitric acid.

#### 3.3.3. Enrichment Analysis of Core Metabolic Pathways

**Figure 4:**
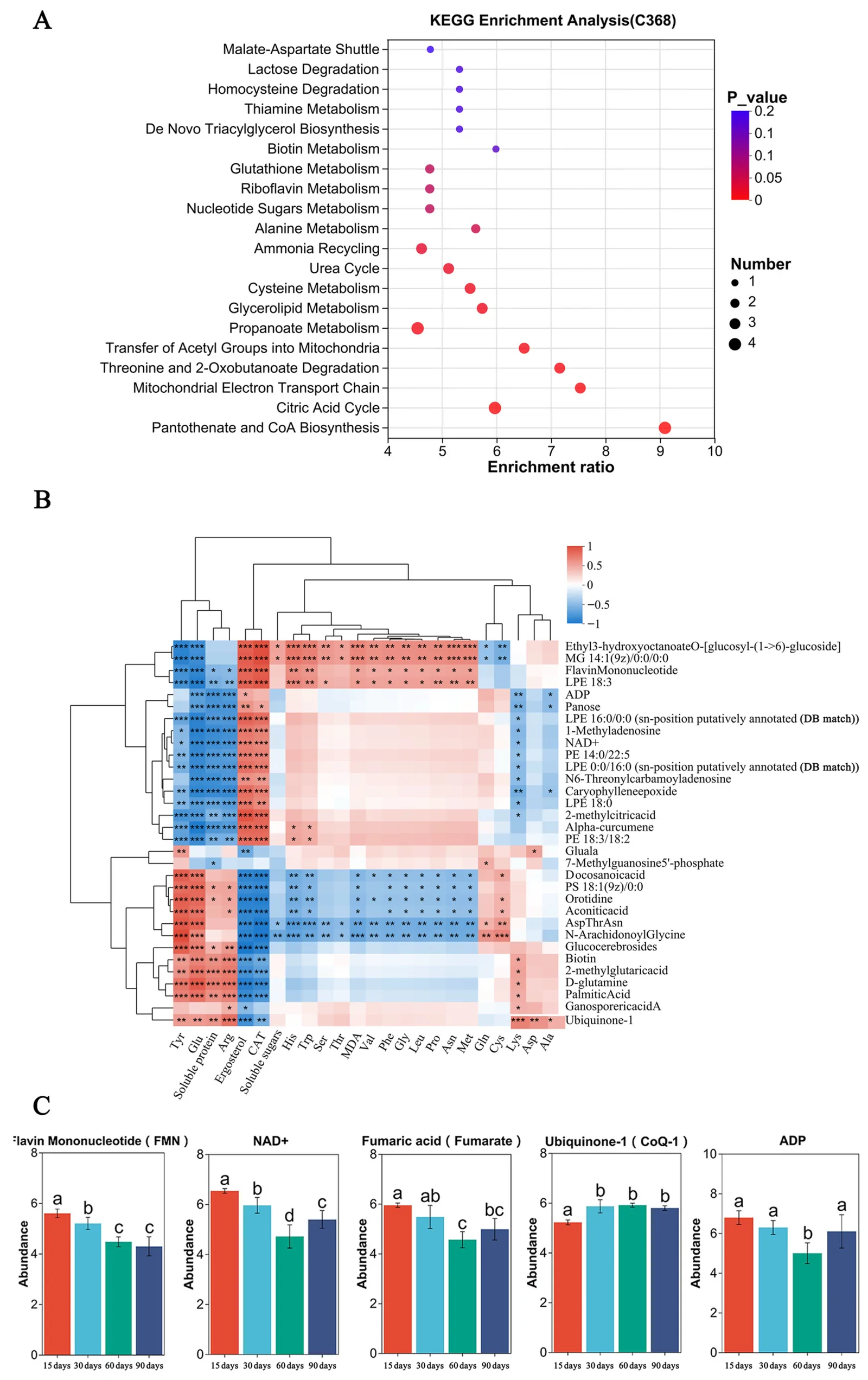
Functional analysis and correlation of the core metabolites of C368. (**A**) KEGG enrichment pathway analysis of C368, (**B**) Correlation analysis between core metabolites of C368 and physiological-biochemical indicators. *p*-values marked as * (0.01 < *p* ≤ 0.05), ** (0.001 < *p* ≤ 0.01), or *** (*p* ≤ 0.001), (**C**) Relative metabolite concentrations of five core metabolites in the oxidative phosphorylation pathway.

To further analyze metabolite changes, KEGG pathway enrichment analysis was carried out. Metabolite sets from C104 and C317 were merged to form the core metabolite set C368. KEGG pathway enrichment analysis was performed (Figure 4A and Table S5), and a core metabolite–pathway network was constructed (Figure S1). Pathways with *p*_adjust < 0.05 included oxidative phosphorylation; cutin, suberin, and wax biosynthesis; biosynthesis of cofactors; and arachidonic acid metabolism. The network association diagram shows that the significantly enriched pathways were divided into three network groups, among which the network comprising oxidative phosphorylation and the biosynthesis of cofactors had the highest density. Within this network, oxidative phosphorylation links five core metabolites: flavin mononucleotide, NAD^+^, fumarate, ubiquinone-1, and ADP; the biosynthesis of cofactors is linked to flavin mononucleotide, NAD^+^, ubiquinone-1, and ADP; Nicotinate and nicotinamide metabolism was linked to NAD^+^ and fumarate; tyrosine metabolism and the TCA cycle were linked to fumarate; and thiamine metabolism was linked to NAD^+^. Furthermore, L-cysteine was linked to nine pathways, including thiamine metabolism and the biosynthesis of cofactors, making it the core metabolite with the most linked pathways.

#### 3.3.4. Correlation Analysis Between Core Metabolites and Physiological-Biochemical Indicators

C104 and C317 were combined to form the core metabolite set Core368 (C368), and an association analysis was performed between C368 and physiological-biochemical indicators (the correlation coefficient is denoted by R) (Figure 4B). Correlation analysis of the core metabolite set C368 with physiological-biochemical indicators revealed that MG 14:1_0:0_0:0 showed a strong positive correlation with CAT activity (R = 0.944) and a strong negative correlation with tyrosine (R = −0.892); aspartic acid-threonine-aspartamide showed a strong negative correlation with CAT (R = −0.938) and a strong positive correlation with tyrosine (R = 0.882); NAD^+^ was positively correlated with ergosterol and CAT and negatively correlated with MDA. A highly synergistic pattern of association exists between core metabolites and antioxidant enzymes, membrane lipid peroxidation products, and specific amino acids.

As the culture age increased, among the five core metabolites of the oxidative phosphorylation pathway, Flavin mononucleotide (FMN) expression showed a decreasing trend, while CoQ-1 expression initially increased and then stabilized. NAD^+^, fumarate, and ADP exhibited similar trends, initially decreasing and then increasing, with the inflection point occurring at 60 days for all three (Figure 4C).

#### 3.3.5. Analysis of the Structure of the Mycelium-Associated Bacterial Community

**Figure 5:**
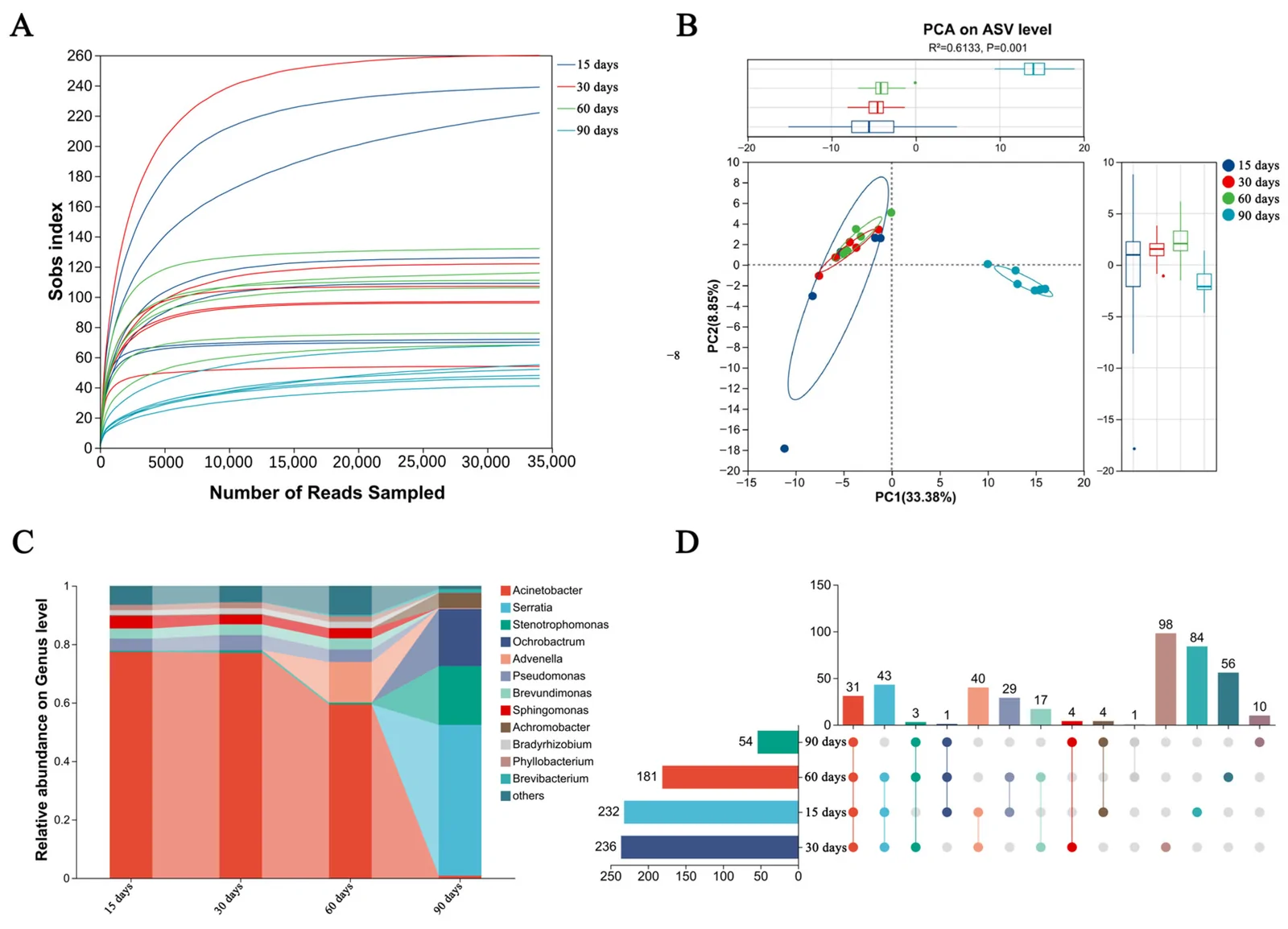
Analysis of the structure of the mycelium-associated bacterial community. (**A**) Rarefaction curve, (**B**) Principal component analysis, (**C**) Community composition analysis.The *x*-axis and *y*-axis denote samples of different mycelial ages and species relative abundance. Colored bars represent different species, and bar length reflects species proportion, (**D**) Upset analysis. The left horizontal bar chart shows element counts of each sample group. Dots in the central matrix stand for group-specific elements, and connecting lines represent shared elements. The right vertical bar chart indicates the quantity of intersecting elements.

All 16S rRNA sequencing samples in mycelium-associated bacteria from *F. filiformis* mycelia at different ages (15, 30, 60 and 90 days) were flattened to a minimum sample sequence number of 34,046, and a total of 1374 ASVS were annotated, covering 32 phyla, 71 classes, 162 orders, 242 families, 421 genera, and 515 species. In order to further verify whether the number of sequences after flattening reaches the sequencing depth, the study draws the dilution curve with the observed number of species (Sobs index) as the ordinate and the number of sequences as the abscissa. The dilution curve in each sample tends to be flat after the number of sequences exceeds 30,000 (Figure 5A). Therefore, the number of sequences has reached the sequencing depth and can be analyzed in the next step.

At the genus level, *Acinetobacter* was the dominant genus in the 15-, 30-, and 60-day groups, with relative abundances of 77.33%, 77.02%, and 59.50%, respectively. In the 90-day group, the abundance of *Acinetobacter* declined sharply to 0.84%, while *Serratia* (51.57%), *Stenotrophomonas* (20.13%), and *Ochrobactrum* (19.53%) became the dominant genera (Figure 5C). The species richness of mycelium-associated bacteria showed a decreasing trend with increasing culture duration: 15, 30, 60, and 90 days shared 31 genera; 15, 30, and 60 days shared 43, while 30, 60, and 90 days shared only 3. The number of genera endemic to 15, 30, 60, and 90 days was 84, 98, 56, and 10, respectively (Figure 5D).

Based on upset analysis and ASV annotation data, this study further identified core microorganisms within bacterial communities at the genus level. Genuses with a presence rate of ≥90% and an abundance share of ≥0.1% were defined as core genera, and their abundance shares were calculated (with the abundance of all genera in the sample serving as the denominator). The signature genera in the 15-day group were *Acinetobacter* and *Sphingomonas*; *Pseudomonas* and *Phyllobacterium* in the 30-day group; *Brevundimonas* and *Bradyrhizobium* in the 60-day group; *Stenotrophomonas* and *Achromobacter* in the 90-day group (Table S6). The phasic succession of these signature genera closely corresponded with the mycelial ageing process.

#### 3.3.6. Correlation Analysis

To reveal potential associations between physiological-biochemical indicators, core metabolites and the mycelium-associated bacterial community, a systematic correlation analysis was conducted in this study.

**Figure 6:**
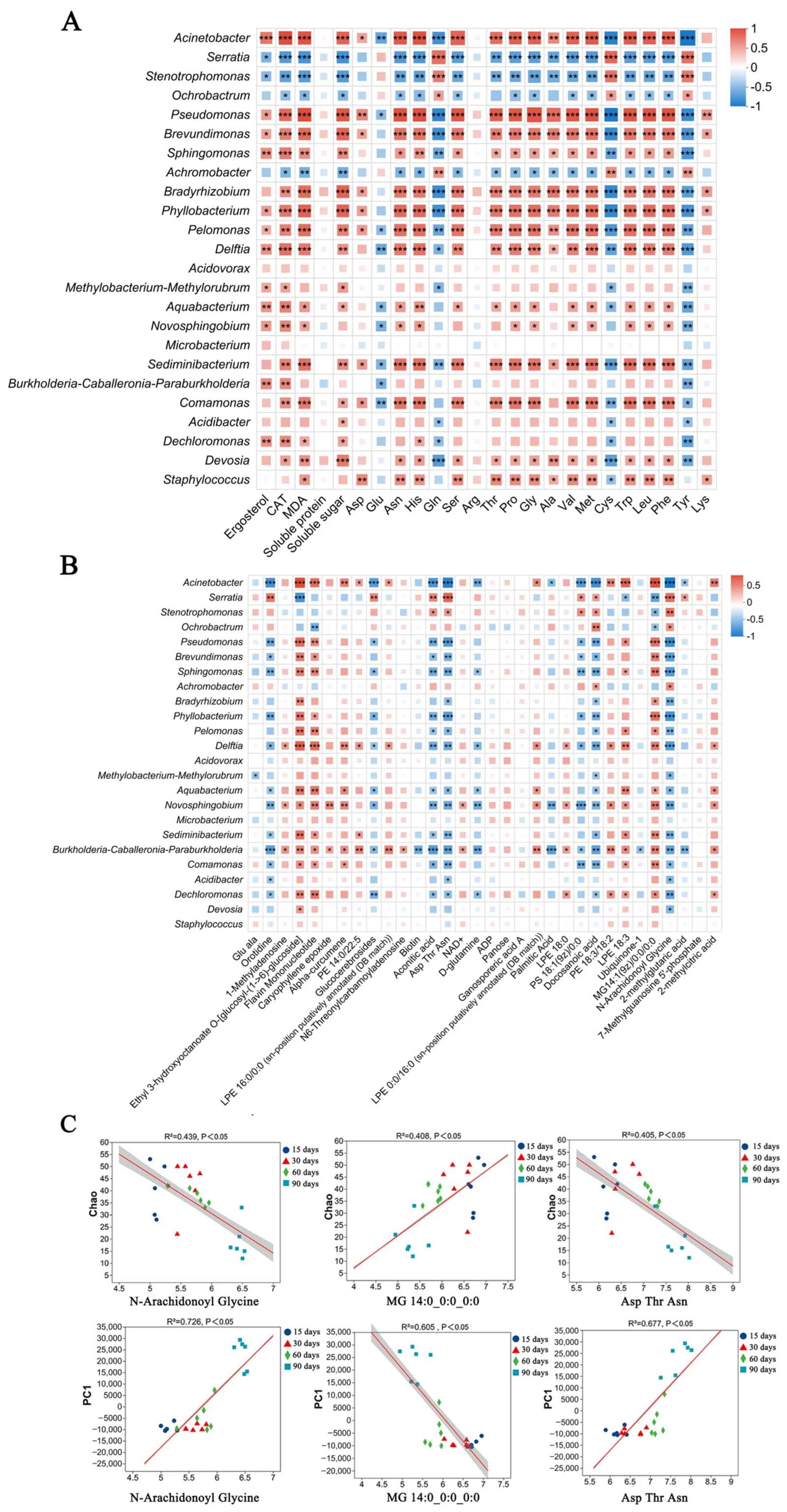
Correlation analysis. (**A**) Heat map of correlations between core bacterial genera and physiological indicators. X/Y axes show physiological indicators/core genera, with R-values color-coded (see legend). *p*-values marked as * (0.01 < *p* ≤ 0.05), ** (0.001 < *p* ≤ 0.01), or *** (*p* ≤ 0.001), (**B**) Heat map of correlations between core bacterial genera and core metabolites of C368. X/Y axes show key metabolites/core genera, with R-values color-coded (see legend). *p*-values marked as * (0.01 < *p* ≤ 0.05), ** (0.001 < *p* ≤ 0.01), or *** (*p* ≤ 0.001), (**C**) Regression analysis. Panels in the first row display ranked regression between factors and the Chao index, whereas Panels in the second row show ranked regression between factors and PC1. The *x*-axis includes N-Arachidonoyl glycine, MG 14:1_0:0_0:0, and Asp Thr Asn. The *y*-axis represents the Chao index and PC1. Higher R2 implies greater explanatory power of the factor for sample differences. The red line represents the fitted linear regression line, and the shaded grey area indicates the 95% confidence interval of the regression.

Correlation analysis of core bacterial genera with physiological-biochemical indicators revealed that *Acinetobacter* showed a significant positive correlation with CAT, ergosterol and soluble sugars (with R values of 0.871, 0.675, and 0.764, respectively), and a significant negative correlation with MDA and tyrosine. Conversely, *Serratia* showed significant negative correlations with CAT, ergosterol and soluble sugars (R = −0.658, −0.492, and −0.716, respectively), and positive correlations with MDA and tyrosine. Tyrosine was significantly correlated with 21 core bacterial genera, making it the physiological-biochemical indicator associated with the greatest number of genera. No significant correlation was observed between soluble proteins and the core bacterial genera (Figure 6A).

An analysis of the correlation between core bacterial genera and core metabolites revealed that N-arachidonyl glycine was significantly correlated with 21 core bacterial genera, showing a positive correlation with the genus *Serratia* (R = 0.630) and a negative correlation with the genus *Acinetobacter* (R = −0.874). MG 14:1_0:0_0:0 was positively correlated with *Acinetobacter* (R = 0.783) and negatively correlated with *Serratia* (R = −0.605). Aspartic acid-threonine-asparagine showed a positive correlation with the *Serratia* genus (R = 0.680) and a negative correlation with the *Acinetobacter* genus (R = −0.819). Docosanoic acid showed a negative correlation with the *Acinetobacter* genus (R = −0.728) and a positive correlation with the *Ochrobactrum* genus (R = 0.575). FMN was positively correlated with the genus *Delftia* (R = 0.645) and negatively correlated with the genus *Ochrobactrum* (R = −0.542) (Figure 6B).

Hierarchical regression analysis was used to assess the explanatory power of core metabolites on bacterial community diversity. Linear regression was performed with the Chao 1 index and principal component PC1 as dependent variables, and the expression levels of N-arachidonyl glycine, MG 14:1_0:0_0:0, and aspartic acid-threonine-aspartamide as independent variables. The results showed that N-arachidonyl glycine exhibited the highest explanatory power for both the Chao 1 index (R^2^ = 0.439) and PC1 (R^2^ = 0.726), indicating that this metabolite is the primary factor driving or responding to changes in the structure of the mycelium-associated bacterial community. MG 14:1_0:0_0:0 and aspartic acid-threonine-aspartamide also exhibited significant explanatory power (*p* < 0.05) (Figure 6C).

## 4. Discussion

Analysis of physiological and biochemical indicators revealed that the levels of soluble proteins, soluble sugars, and most free amino acids (such as leucine, threonine, and valine) followed an initial upward trend followed by a decline, suggesting that stress-induced metabolic upregulation may occur during the early stages of mycelial ageing, with a subsequent transition to a phase of metabolic decline. Ergosterol content and CAT activity declined continuously with increasing mycelial age, decreasing by 50% and 77.78%, respectively, at 90 days compared with 15 days.

The decrease in ergosterol content reflects impaired membrane structural integrity [36,37]; the decline in CAT activity indicates reduced antioxidant capacity [38], which may be associated with dysregulation of the autophagy-mitophagy transition in peroxisomes and mitochondria [39]. MDA levels exhibited an initial rise followed by a decline. During the early stages of fungal growth, when metabolism is vigorous, the production of reactive oxygen species (ROS) may increase, leading to intensified lipid peroxidation; as MDA is a final product of this process, its levels consequently rise. The subsequent decrease in later stages may be related to severe disruption of cellular structure and a reduction in substrates for lipid peroxidation during the late stages of ageing [40,41]. Leucine has been shown to delay yeast ageing via pathways such as TOR activation [38]; its concentration decreased by 82.43% at 90 days, which may be associated with accelerated mycelial ageing. Conversely, the continuous rise in tyrosine levels may indicate activation of the melanin synthesis pathway [42], and the accumulation of the secondary metabolite melanin suggests that the mycelia have entered the ageing phase.

The results of the mycelial morphological observations are consistent with previous studies. The swelling of the mycelia may be related to the enlargement of vacuoles. As vacuoles serve as the primary sites for autophagy and material degradation [43], any dysfunction in their function can lead to the shriveling of the mycelia and the leakage of their contents. The fruiting results indicate that fruiting performance begins to decline at 30 days, reaches a turning point between 60 and 75 days, and is completely lost by 90 days. The above time points can serve as important reference indicators for evaluating the viability of *F. filiformis* strains.

The oxidative phosphorylation pathway was significantly enriched across multiple comparison groups, with its significance decreasing markedly as fungal age increased, suggesting that oxidative phosphorylation may be one of the intrinsic drivers of mycelial ageing. Day 60 marked the inflection point at which the significance of oxidative phosphorylation in mycelia declined and the expression levels of multiple core metabolites changed, indicating that 60 days is a critical juncture for changes in metabolic characteristics. The signature metabolites identified in both C104 and C317 were aspartate-threonine-aspartamide, NAD^+^, and MG 14:1_0:0_0:0; these three metabolites can serve as important signature metabolites reflecting the metabolic characteristics of mycelia. NAD^+^ has been shown to delay ageing in yeast [44]. MG 14:1_0:0_0:0 belongs to the glycerolipids. Research by Camille et al. [45] indicates that MG can enhance and maintain mitochondrial oxidative capacity, thereby delaying ageing in fruit flies. The content of MG 14:1_0:0_0:0 shows a strong positive correlation with CAT activity, suggesting that it may be involved in antioxidant processes, which is consistent with the function of monoglycerides [46]. Aspartate-threonate-aspartamide showed a strong negative correlation with CAT activity; tripeptides such as glutathione [47] play an important role in anti-ageing, and studies have shown that the isomerization of aspartate and aspartamide is a marker of cellular ageing and protein degradation in organisms [48]. NAD^+^, MG 14:1_0:0_0:0 and aspartic acid-threonine-aspartamide all have the potential to serve as ageing marker metabolites.

Microbiomics analysis revealed that the mycelium-associated bacterial community underwent a fundamental shift at 90 days, with the dominant genus changing from *Acinetobacter* to *Serratia*. This is not the first report of the genus *Serratia* in edible fruiting body cultivation systems. *Serratia odorifera* has also been detected and identified in studies on *Hypsizygus marmoreus*, suggesting that the bacteria may be commonly present in fruiting body cultivation systems [49]. Upset analysis and ASV annotation data further confirmed signature genera at different stages of fungal age, suggesting that microbial community succession is highly synchronized with the ageing process of the mycelium. *Acinetobacter* was positively correlated with CAT, ergosterol, and soluble sugars and negatively correlated with MDA and tyrosine; *Serratia* exhibited the exact opposite correlation pattern, suggesting that the shift in community structure from *Acinetobacter* to *Serratia* dominance could serve as one of the indicators for assessing the ageing of *F. filiformis*. N-arachidonyl glycine, MG 14:1_0:0_0:0, and aspartic acid -threonine-aspartamide were all effective in assessing the alpha diversity and principal component analysis of bacterial communities, with N-arachidonyl glycine demonstrating the best explanatory power. This suggests that metabolites may be involved in regulating the composition of the mycelium-associated bacterial community or that changes in the microbial community may in turn influence the metabolic profile of the mycelium. Research by Luo et al. [50] indicates that fatty acids in edible fungi, such as docosanoic acid, possess certain antioxidant properties. GML, a member of the MG family, has demonstrated antibacterial activity against *Acinetobacter baumannii* [51]. Since MGs share a common membrane-disrupting mechanism and the unsaturated acyl chain of MG 14:1_0:0_0:0 may further modulate membrane interaction, we hypothesize that MG 14:1_0:0_0:0 may also exhibit activity against *Acinetobacter*, although this requires direct validation. Through combined analysis, the potential of N-arachidonyl glycine and docosanoic acid as ageing-marker metabolites was identified, and the roles of MG 14:1_0:0_0:0 and aspartic acid-threonine-aspartamide as ageing-marker metabolites were further validated.

## Data Availability

The original contributions presented in this study are included in the article/Supplementary Materials. Further inquiries can be directed to the corresponding authors.

## Supplementary Materials

The following supporting information can be downloaded at: https://www.mdpi.com/article/10.3390/jof12090708/s1, Table S1: Nutritional components, membrane homeostasis, and oxidative damage indicators; Table S2: Free amino acids content (Unit: mg·g^−1^); Table S3: Mycelia morphology evaluation; Table S4: Agronomic characteristics; Table S5: C368 KEGG pathway enrichment statistics; Table S6-1: 15 days core bacterial genera; Table S6-2: 30 days core bacterial genus; Table S6-3: 60 days core bacterial genus; Table S6-4: 90 days core bacterial genus; Figure S1: Network association diagram of C368.
