# Supplementary material for: Physio-Morphological Assessment and Multi-Omics Analysis of Flammulina filiformis Mycelial Ageing
Source: J Fungi (Basel). 2026 Sep 21;12(9):708. doi: 10.3390/jof12090708 (PMC13608769; doi:10.3390/jof12090708)
Supplement: Supplementary file 1 [file jof-12-00708-s001.zip › jof-4534469-supplementary.pdf]

Table S1. Nutritional components, membrane homeostasis and oxidative damage indicators.

Sample	Soluble Protein $mg\cdot g^{-1}$	Soluble Sugar $mg\cdot g^{-1}$	Ergosterol Content $mg\cdot g^{-1}$	MDA Content $nmol\cdot g^{-1}$	CAT Activity $U\cdot g^{-1}$
15 days	15.14±1.37^{b}	66.39±3.84^{d}	1.08±0.02^{a}	8.25±0.68^{ab}	2.79±0.90^{a}
30 days	17.00±1.98^{b}	78.68±2.25^{b}	0.73±0.01^{b}	9.84±0.75^{a}	2.22±1.02^{ab}
45 days	24.97±3.49^{a}	88.46±2.57^{a}	0.71±0.02^{b}	10.29±1.23^{a}	1.13±0.51^{bc}
60 days	23.16±3.65^{a}	91.84±4.07^{a}	0.68±0.04^{b}	8.82±0.74^{a}	1.57±0.40^{abc}
75 days	22.59±3.49^{a}	61.33±1.82^{c}	0.56±0.04^{c}	7.99±0.82^{ab}	1.49±0.65^{bc}
90 days	16.56±1.90^{b}	30.47±0.59^{e}	0.54±0.01^{c}	6.12±0.77^{b}	0.62±0.31^{c}

Note: The data in the table are presented as Mean ± SD. Different lowercase letters indicate significant differences ($P < 0.05$).

Table S2. Free amino acids content (Unit: $mg\cdot g^{-1}$).

Amino Acid	15 Days	30 Days	45 Days	60 Days	75 Days	90 Days
Asp	0.234±0.012^{c}	0.417±0.011^{a}	0.243±0.028^{c}	0.341±0.004^{b}	0.185±0.001^{d}	0.241±0.003^{c}
Glu	1.052±0.023^{c}	1.060±0.025^{c}	0.926±0.136^{d}	1.428±0.017^{a}	1.179±0.015^{b}	1.354±0.024^{a}
Asn	0.388±0.007^{c}	0.568±0.012^{a}	0.322±0.038^{d}	0.425±0.005^{b}	0.211±0.001^{e}	0.207±0.001^{e}
His	0.290±0.096^{b}	0.392±0.005^{a}	0.243±0.005^{d}	0.280±0.001^{c}	0.185±0.002^{e}	0.142±0.003^{f}
Gln	0.837±0.006^{b}	0.781±0.009^{c}	0.580±0.063^{e}	0.646±0.005^{d}	0.648±0.003^{d}	1.072±0.014^{a}
Ser	0.598±0.009^{c}	0.960±0.016^{a}	0.523±0.054^{d}	0.740±0.005^{b}	0.371±0.002^{e}	0.340±0.002^{e}
Arg	1.253±0.027^{e}	2.026±0.026^{c}	2.233±0.202^{b}	2.525±0.017^{a}	2.161±0.013^{bc}	1.660±0.009^{d}
Thr	0.336±0.002^{c}	0.711±0.016^{a}	0.253±0.018^{d}	0.474±0.003^{b}	0.174±0.0.0^{e}	0.138±0.009^{f}
Pro	0.327±0.001^{c}	0.547±0.002^{a}	0.293±0.001^{d}	0.368±0.000^{b}	0.179±0.000^{e}	0.149±0.000^{f}
Gly	0.328±0.002^{c}	0.519±0.004^{a}	0.292±0.009^{d}	0.375±0.001^{b}	0.187±0.001^{e}	0.173±0.004^{f}
Ala	0.813±0.019^{c}	1.168±0.030^{b}	0.811±0.117^{c}	1.303±0.016^{a}	0.616±0.005^{d}	0.624±0.006^{d}
Val	0.487±0.002^{c}	0.888±0.010^{a}	0.406±0.005^{d}	0.595±0.000^{b}	0.232±0.000^{e}	0.179±0.000^{f}
Met	0.276±0.003^{c}	0.413±0.008^{a}	0.243±0.004^{d}	0.300±0.006^{b}	0.163±0.005^{e}	0.143±0.005^{f}
Cys	0.196±0.001^{d}	0.172±0.000^{e}	0.213±0.003^{b}	0.168±0.000^{f}	0.204±0.000^{c}	0.252±0.000^{a}
Trp	0.194±0.001^{b}	0.282±0.002^{a}	0.162±0.007^{c}	0.194±0.001^{b}	0.111±0.001^{d}	0.100±0.001^{e}
Leu	0.643±0.004^{c}	1.150±0.013^{a}	0.538±0.018^{d}	0.768±0.002^{b}	0.299±0.000^{e}	0.202±0.000^{f}
Phe	0.537±0.003^{c}	0.844±0.005^{a}	0.482±0.007^{d}	0.623±0.004^{b}	0.281±0.000^{e}	0.266±0.001^{f}
Tyr	0.727±0.014^{e}	1.015±0.057^{c}	0.815±0.067^{d}	1.043±0.005^{c}	1.171±0.004^{b}	1.537±0.009^{a}
Lys	0.988±0.025^{c}	1.814±0.162^{a}	1.496±0.286^{b}	1.852±0.008^{a}	1.344±0.004^{b}	1.095±0.004^{c}

Note: The data in the table are presented as Mean ± SD. Different lowercase letters indicate significant differences ($P < 0.05$).

Table S3. Mycelia morphology evaluation.

Sample	Clamp-Connection	Sporulation Capacity	Aerial Mycelial Strength	Mycelial Density	Mycelial Sectoring
15 days	+++	+	++	++	+
30 days	++	+++	+++	+++	+
45 days	+	++	+++	++	+
60 days	+	+	++	++	+
75 days	+	+	+	++	++
90 days	+	+	+	++	+++

Note: +++ is a high degree of expression, ++ is a general degree, and + is a low degree.

Table S4. Agronomic characteristics.

Sample	Stipe Length mm	Stipe Diameter mm	Pileus Diameter mm	Pileus Height mm	Yield Per Bottle g	Biological Efficiency %
15 days	136.10±15.95^{a}	2.87±0.43^{c}	5.90±1.16^{b}	3.29±0.70^{b}	53.27±2.88^{a}	60.88±3.29^{a}
30 days	121.95±12.96^{b}	3.14±0.62^{b}	6.32±1.41^{a}	3.78±0.60^{a}	51.39±2.98^{b}	56.09±3.40^{b}
45 days	114.04±10.99^{c}	3.39±0.55^{a}	6.32±1.68^{a}	3.29±0.73^{b}	47.40±2.96^{c}	54.16±3.39^{c}
60 days	97.00±9.12^{d}	2.82±0.42^{c}	5.10±0.90^{c}	2.85±0.40^{c}	44.25±0.81^{d}	50.58±0.92^{d}
75 days	70.44±8.49^{e}	3.15±0.64^{b}	5.25±0.93^{c}	3.29±0.53^{b}	42.88±1.27^{e}	49.00±1.45^{e}

Note: The data in the table are presented as mean ± standard deviation (Mean ± SD). Different lowercase letters indicate significant differences ($P < 0.05$).

Table S5. C368 KEGG pathway enrichment statistics.

Pathway Description	Pathway ID	Metabolites Number	Enrich Factor	P_value	P_adjust
Oxidative phosphorylation	map00190	5	31.25%	3.664×10^{-8}	1.942×10^{-6}
Cutin, suberine and wax biosynthesis	map00073	3	11.11%	0.0007	0.0175
Biosynthesis of cofactors	map01240	8	2.43%	0.0010	0.0183
Arachidonic acid metabolism	map00590	4	5.06%	0.0016	0.0212
Glycerophospholipid metabolism	map00564	3	5.36%	0.0055	0.0582
Biosynthesis of unsaturated fatty acids	map01040	3	4.05%	0.0119	0.1022
Pantothenate and CoA biosynthesis	map00770	2	6.67%	0.0158	0.1022
Thiamine metabolism	map00730	2	6.45%	0.0169	0.1047
Carbapenem biosynthesis	map00332	2	6.25%	0.0179	0.1054
Autophagy - other	map04136	1	33.33%	0.0193	0.1117
Autophagy - yeast	map04138	1	33.33%	0.0193	0.1199
Cyanoamino acid metabolism	map00460	2	4.44%	0.0339	0.1497
Glycosylphosphatidylinositol (GPI)-anchor biosynthesis	map00563	1	14.29%	0.0444	0.1811
Nicotinate and nicotinamide metabolism	map00760	2	3.64%	0.0489	0.1849

Table S6-1. 15 days core bacterial genera.

Number	Genus	Prevalence	Percent
1	*Acinetobacter*	100.00%	75.99%
2	*Sphingomonas*	100.00%	4.20%
3	*Pseudomonas*	100.00%	4.08%
4	*Brevundimonas*	100.00%	3.42%
5	*Phyllobacterium*	100.00%	1.80%
6	*Bradyrhizobium*	100.00%	1.66%
7	*Pel/omonas*	100.00%	0.69%
8	*Delftia*	100.00%	0.57%
9	*Methylobacterium-Methylorubrum*	100.00%	0.34%
10	*Burkholderia-Caballeronia-Paraburkholderia*	100.00%	0.28%
11	*Aquabacterium*	100.00%	0.28%
12	*Novosphingobium*	100.00%	0.27%
13	*Dechloromonas*	100.00%	0.14%
14	*Acidibacter*	100.00%	0.11%

Table S6-2. 30 days core bacterial genus.

Number	Genus	Prevalence	Percent
1	*Acinetobacter*	100.00%	75.77%
2	*Pseudomonas*	100.00%	5.08%
3	*Brevundimonas*	100.00%	3.64%
4	*Sphingomonas*	100.00%	3.15%
5	*Phyllobacterium*	100.00%	2.01%
6	*Bradyrhizobium*	100.00%	1.93%
7	*Pelomonas*	100.00%	0.91%
8	*Stenotrophomonas*	100.00%	0.74%
9	*Sediminibacterium*	100.00%	0.26%
10	*Novosphingobium*	100.00%	0.24%
11	*Comamonas*	100.00%	0.21%
12	*Burkholderia-Caballeronia-Paraburkholderia*	100.00%	0.18%
13	*Acidibacter*	100.00%	0.10%

Table S6-3. 60 days core bacterial genus.

Number	Genus	Prevalence	Percent
1	*Acinetobacter*	100.00%	58.79%
2	*Pseudomonas*	100.00%	4.24%
3	*Brevundimonas*	100.00%	3.76%
4	*Sphingomonas*	100.00%	3.39%
5	*Bradyrhizobium*	100.00%	2.13%
6	*Phyllobacterium*	100.00%	1.89%
7	*Pelomonas*	100.00%	0.64%
8	*Stenotrophomonas*	100.00%	0.60%
9	*Delftia*	100.00%	0.37%
10	*Methylobacterium-Methylorubrum*	100.00%	0.33%
11	*Acidovorax*	100.00%	0.25%
12	*Aquabacterium*	100.00%	0.16%
13	*Sediminibacterium*	100.00%	0.15%
14	*Achromobacter*	100.00%	0.12%
15	*Devosia*	100.00%	0.11%
16	*Microbacterium*	100.00%	0.10%

Table S6-4. 90 days core bacterial genus.

Number	Genus	Prevalence	Percent
1	*Serratia*	100.00%	51.56%
2	*Stenotrophomonas*	100.00%	20.11%
3	*Ochrobactrum*	100.00%	19.53%
4	*Achromobacter*	100.00%	5.20%
5	*Acinetobacter*	100.00%	0.84%

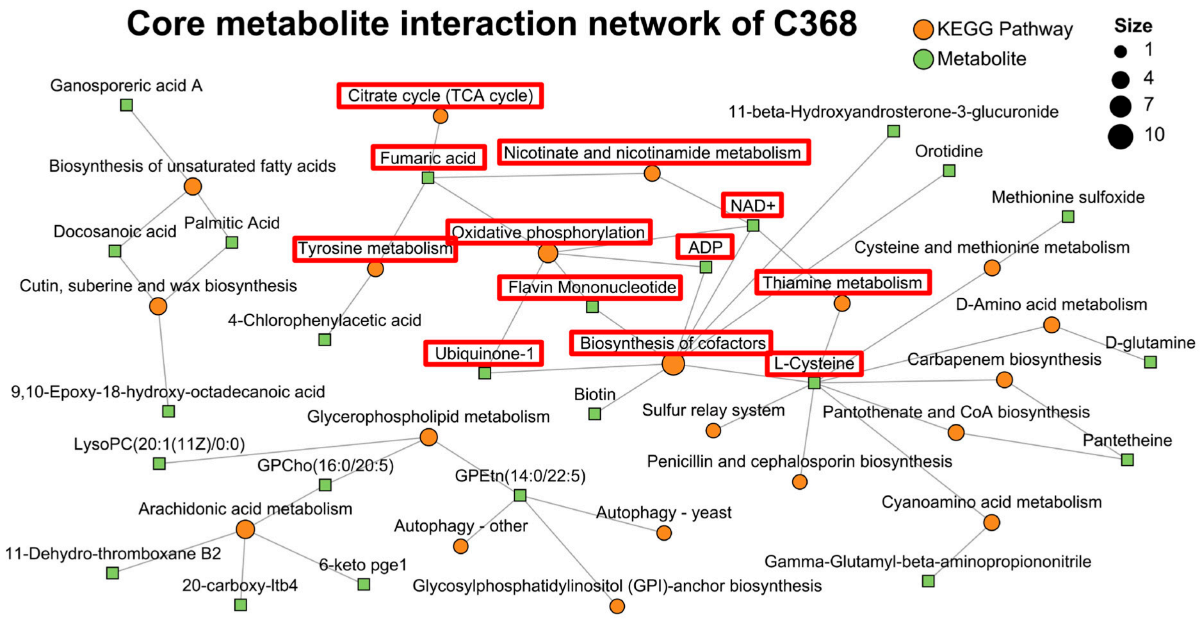

Figure S1. Network association diagram of C368.
